# Survival time and prognostic factors after whole-brain radiotherapy of brain metastases from of breast cancer

**DOI:** 10.1177/2058460120938744

**Published:** 2020-07-06

**Authors:** Yukinori Okada, Mariko Kobayashi, Mio Shinozaki, Tatsuyuki Abe, Yoshihide Kanemaki, Naoki Nakamura, Yasuyuki Kojima

**Affiliations:** 1 Department of Radiology, St. Marianna University School of Medicine, Kawasaki, Japan; 2 Department of Surgery, Division of Breast and Endocrine Surgery, St. Marianna University School of Medicine, Kawasaki, Japan

**Keywords:** Breast cancer, brain metastasis, whole-brain radiotherapy

## Abstract

**Background:**

Breast cancer has a poor prognosis due to the high risk of distant metastasis.

**Purpose:**

To identify the prognosticators of brain metastasis from breast cancer treated by whole-brain radiotherapy.

**Material and Methods:**

We evaluated patients diagnosed with primary brain metastasis without carcinomatous meningitis from breast cancer and had undergone whole-brain radiotherapy as initial treatment between 1 January 2010 and 30 September 2019. We investigated associations between overall survival time from diagnosis using cranial contrast-enhanced magnetic resonance imaging (MRI)/computed tomography (CT) and the following parameters: (i) age; (ii) sex; (iii) time to appearance of brain metastasis; (iv) other metastasis at appearance of brain metastasis; (v) blood test; (vi) symptoms at time of brain metastasis; (vii) whole-brain radiotherapy dose; (viii) whether whole-brain radiotherapy was completed; (ix) course of chemo- or radiotherapy; (x) subtype; (xi) additional irradiation after whole-brain radiotherapy; (xii) pathology; and (xiii) imaging findings.

**Results:**

We evaluated 29 consecutive female patients (mean age 55.2 ± 12.1 years). Median overall survival time after diagnosis on cranial contrast-enhanced MRI/CT was 135 days (range 16–2112 days). Multivariate stepwise analysis of the three parameters of lactate dehydrogenase, dose, and subtype identified the following significant differences: Hazard Ratio (HR) for dose (discontinued, 30 Gy/10 fractions, 31.5 Gy/11 fractions, 32.5 Gy/11 fractions, 37.5 Gy/15 fractions) was 0.08 (95% confidence interval [CI] 0.02–0.30, *P* < 0.01), and HR for subtype (luminal, HER2, triple-negative) was 2.70 (95% CI 1.16–6.243, *P* < 0.01).

**Conclusion:**

HER2-type and 37.5 Gy/15 fractions are good prognostic factor after whole-brain radiotherapy in breast cancer with brain metastases.

## Introduction

Breast cancer is the most common malignant tumor among Japanese women (1). In around 25% of cases, patients with breast cancer develop brain metastases (2). One study found that brain metastasis was already present at the time of initial examination in 7.2% of patients and developed later in 17.5% (2).

When brain metastasis is identified, treatment is usually provided in the form of radiotherapy. Radiotherapy for brain metastasis can be classified into stereotactic radiotherapy or whole-brain radiotherapy. In patients with a single brain metastasis, the addition of whole-brain radiotherapy to stereotactic radiotherapy has been found to prolong time of overall survival (OS) (3). In patients with two or three metastases, however, addition of whole-brain radiotherapy to stereotactic radiotherapy appears to have no effect on prolonging time of OS (3). Such patients may therefore be treated with stereotactic radiotherapy alone and kept under careful observation.

Other studies have found that in patients with 1–3 brain metastases, addition of whole-brain radiotherapy to stereotactic radiotherapy or surgery did not improve OS time (4), and no improvement was apparent in the survival of patients with three brain metastases who underwent whole-brain radiotherapy in addition to stereotactic radiotherapy (5).

Yet another study found that in patients aged ≤50 years with 1–4 brain metastases, addition of whole-brain radiotherapy to stereotactic radiotherapy did not improve OS time (6). If ≤10 brain metastases are present, there is reportedly scope to avoid whole-brain radiotherapy in favor of stereotactic radiotherapy and careful follow-up (7). Underlying this policy of avoiding whole-brain radiotherapy is the fact that cognitive function frequently declines 2–3 years after the conclusion of whole-brain radiotherapy (8).

Other studies have also reported high frequencies of cognitive impairment after whole-brain radiotherapy (9,10). The frequency of cognitive impairment was reported to be significantly lower among long-term survivors who had undergone stereotactic radiotherapy alone than among those who had undergone both whole-brain and stereotactic radiotherapy (11). Given that brain metastasis to the hippocampus is rare (12) and declines in cognitive function are believed to stem from apoptosis of cells in the hippocampus, the use of intensity-modulated radiotherapy (IMRT) in whole-brain radiotherapy to reduce the dose to the hippocampus has also been considered, and cognitive function with this technique is comparatively conserved at six months after the conclusion of radiotherapy compared with that after regular whole-brain radiotherapy (13). However, cognitive impairment occurs 2–3 years after the conclusion of whole-brain radiotherapy (8). In addition, cognitive impairment was more frequent within two years of the conclusion of stereotactic radiotherapy alone than after stereotactic radiotherapy plus whole-brain radiotherapy (8). This may be because the progression of brain metastases causes deteriorations in cognitive function, and poorly controlled metastatic brain tumors are another cause of cognitive impairment.

When IMRT is used, radiotherapy planning requires time. In addition, from the perspective of patient prognosis, the question must be considered of whether to avoid whole-brain radiotherapy altogether, or to perform this treatment while proactively reducing the dose to the hippocampus. The aim of the present study was to investigate the treatment outcome of whole-brain radiotherapy for multiple brain metastases from breast cancer as initial treatment and to investigate the prognosis of patients to identify prognostic factors.

## Material and Methods

### Study design

This was a retrospective study conducted at a single center. We investigated the electronic medical records and radiotherapy records of patients with primary brain metastasis without carcinomatous meningitis from breast cancer and had undergone whole-brain radiotherapy as initial treatment between 1 January 2010 and 30 September 2019. The diagnosis of brain metastasis was based on contrast-enhanced computed tomography (CT) or magnetic resonance imaging (MRI). The exclusion criteria were: (i) the progress was unclear; (ii) the number of brain metastases could not be evaluated; (iii) had the few brain metastases with 4 or under in these cases.

### Whole-brain radiotherapy

Whole-brain radiotherapy was administered by bilateral four-field irradiation with 6-MV X-rays using a Primus system (Cannon Medical Systems, Ohtawara, Japan) or Synergy system (Elekta, Stockholm, Sweden). Mevatron (Cannon Medical System, Ohtawara, Japan) was used for extra beam irradiation therapy until 2012.The field-in-field technique was used to attenuate high-dose regions. Gross tumor volume was the volume of tumor identifiable on images, and the clinical target volume (CTV) was the entire brain. Planning target volume was set at a distance of 1.5–2 cm from the CTV. The irradiation field was produced using a 1-cm-width multileaf collimator.

### Survival time

OS time was calculated using the date of diagnosis of brain metastasis on cranial contrast-enhanced MRI/CT as day 1. We investigated associations between OS time and: (i) age; (ii) sex; (iii) time to appearance of brain metastasis; (iv) stage at initial examination; (v) other metastasis on identification of brain metastasis; (vi) blood test results = white blood cell count (WBC), red blood cell count (RBC), platelets, total protein, albumin, lactate dehydrogenase (LDH); C-reactive protein (CRP), carcinoembryonic antigen (CEA), and carbohydrate antigen 15-3 (CA15-3); (vii) symptoms at time of identification of brain metastasis; (viii) whether whole-brain radiotherapy was completed; (ix) course of chemo- or radiotherapy; (x) subtype; (xi) additional irradiation after whole-brain radiotherapy; (xii) pathology; and (xiii) imaging findings (tumor diameter, number of brain metastases).

### Statistical analysis

The date of final follow-up was 30 September 2019, at which point all data were censored. EZR (easy R), developed by Jichi Medical University Saitama Medical center Omiya Hospital) was used for all statistical analyses. OS time was investigated by Kaplan–Meier analysis (log-rank test) and Cox’s proportional hazard model, with values of *P* < 0.05 considered statistically significant.

### Ethical considerations

This study was approved by the Institutional Review Board of St. Marianna University School of Medicine (approval no. 4614). Patients were recruited using the opt-out methodology as provided on the hospital website and in the hospital.

## Results

### Patient selection

#### Number of patients, age, and sex

We enrolled 54 patients; the progress of 15 patients was not clear, the number of brain metastases could not be evaluated in three patients, and seven patients had a low number of brain metastases (≤4). In these cases, stereotactic radiotherapy was considered as the first choice of treatment. We excluded these 25 patients. Finally, 29 consecutive female patients were included (mean age = 55.2 ± 12.1 years).

#### Time to appearance of brain metastasis and stage

In 5 (17%) patients, brain metastasis was already present at initial examination, and all five cases were diagnosed as stage IV. In the other 24 (83%) patients, metastasis developed after the initial examination. In 27 patients, metastasis to another organ was already present when brain metastases were identified.

#### Blood tests

Mean WBC was 8655 ± 5352/µL, with four patients showing the reference value of ≤4000/µL. Mean RBC was 3.7 ± 0.7 × 10^6^/µL, with 14 patients showing the reference value of ≤3.8 × 10^6^/µL. Mean PLT was 26.0 ± 7.7 × 10^4^/µL, with one patient showing the reference value of ≤15.7 × 10^4^/µL. Mean total protein was 6.5 ± 0.9 IU/L, with 15 patients showing the reference value of ≤6.8 IU/L. Mean albumin was 3.4 ± 0.8 g/dL (n = 14, with nine patients showing the reference value of ≤3.9 g/dL. Mean LDH was 359.5 ± 245.1 IU/L (n = 26), with 12 patients showing the reference value of ≤230 IU/L. Mean CRP was 3.7 ± 5.6 g/dL (n = 13), with seven patients showing the reference value of ≤0.3 g/dL. Mean CEA was 14.4 ± 31.9 ng/mL (n = 27), with 13 patients showing the reference value of ≤4.3 ng/mL. Mean CA15-3 was 101.2 ± 157.9 U/mL (n = 27), with nine patients showing the reference value of ≤27 U/mL.

#### Central nervous system symptoms

Neurological symptoms were present when brain metastasis appeared in 11 patients (seizures in three cases, dizziness in two cases, and visual field disturbance, reduced level, headache, weakness, delirium, and paralysis in one case each); there were no symptoms in 18 patients.

#### Dose and completion of treatment

Thirteen patients received 30 Gy/10 fractions, 31.5 Gy/11 fractions, or 32.5 Gy/11 fractions, 14 patients received 37.5 Gy/15 fractions, and two patients discontinued radiotherapy (3 Gy/1 fraction and 15 Gy/5 fractions).

#### Chemotherapy

Chemotherapy was combined with whole-brain radiotherapy in five patients.

#### Subtype

The subtype was the luminal type in eight patients, HER2 in seven patients, triple-negative in 13 patients, and unknown in one patient. Luminal-HER2 type was classified as HER2 type.

#### Additional stereotactic radiotherapy

Stereotactic radiotherapy was performed after whole-brain radiotherapy in three cases.

#### Pathology

The pathology was scirrhous carcinoma in 13 patients, solid tubular carcinoma in seven patients, papillotubular carcinoma in two patients, micropapillary carcinoma in one patient, ductal carcinoma in situ in one patient, and unknown in five patients.

### CT/MRI

The evaluation of brain metastasis was done by contrast-enhanced MRI in 28 patients and contrast enhancement CT in one patient.

The number of brain metastasis was >5 (in those who were subjected to whole-brain radiotherapy). The number of brain metastases was 6–9 in 11 patients and >10 in 18 patients. The maxim size of brain metastasis was >3 cm in three patients. Brain metastases in the cerebellum were detected in 28 patients. Brain metastases in the brainstem were detected in nine patients.

### Survival times

#### Median survival time

Median survival time from diagnosis by cranial contrast-enhanced MRI/CT was 135 days (range = 16–2112 days); 25 patients died during follow-up and four survived to the date of censorship.

#### Kaplan–Meier analysis

The results below were obtained by Kaplan–Meier analysis.

**Fig. 1.**
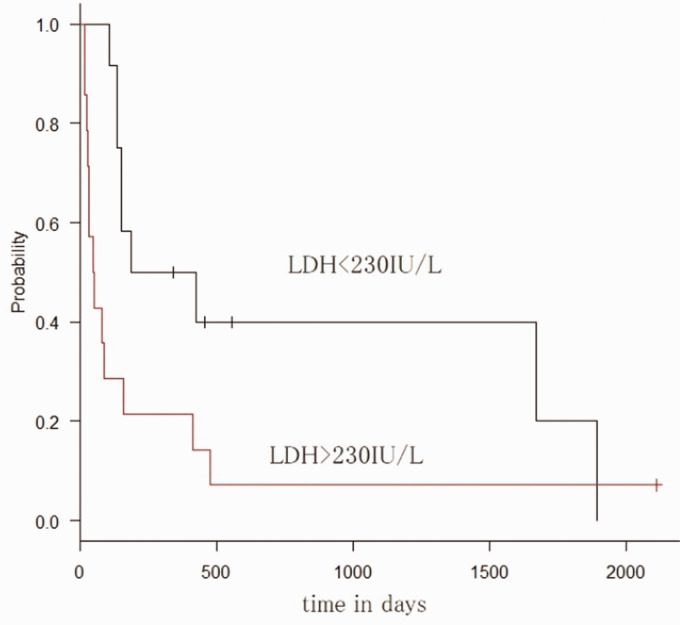
Lactate dehydrogenase and Kaplan–Meier curves.

Median survival time for patients with LDH levels within the reference range was 306 days, compared with 49 days for other patients (*P* = 0.03) (Fig. 1).

**Fig. 2.**
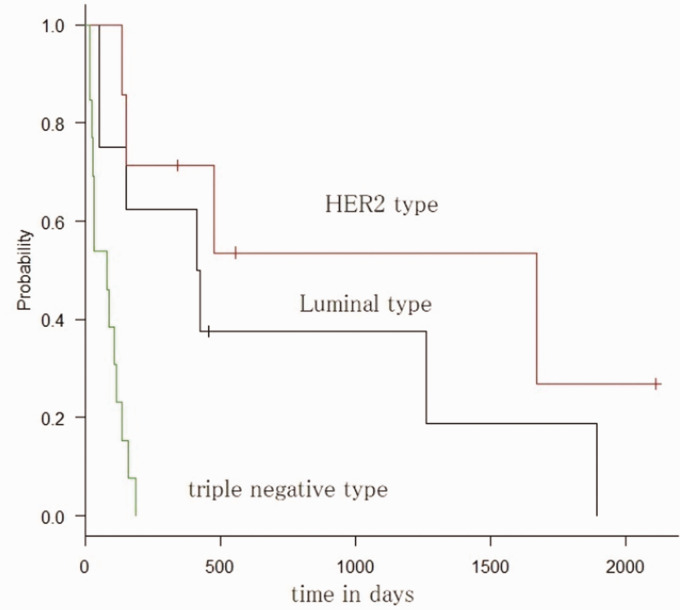
Subtypes and Kaplan–Meier curves.

Median survival time was 419 days for patients with luminal-type breast cancer, 1673 days for those with HER2-type breast cancer, and 80 days for those with triple-negative breast cancer (*P* < 0.01) (Fig. 2).

**Fig. 3.**
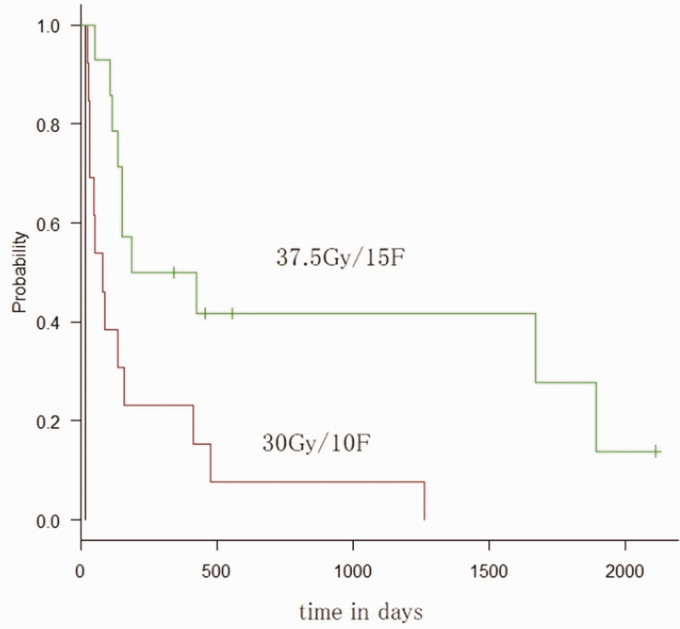
Dose and Kaplan–Meier curves.

Median survival time was 16 days for patients who discontinued whole-brain radiotherapy, 80 days for those who received 30 Gy/10 fractions–31.5 Gy/11 fractions and 32.5 Gy/11 fractions, and 306 days for those who received 37.5 Gy/15 fractions (*P* < 0.01) (Fig. 3). No significant differences in any other factors were identified.

#### Cox’s proportional hazard model

The results below were obtained from Cox’s proportional hazard model.

### Univariate analysis

The hazard ratio (HR) for patients with LDH levels within the reference range compared with those above the reference range was 2.45 (95% confidence interval [CI] = 1.03–5.82, *P* = 0.04). The HR for dose (discontinued, 30 Gy/10 fractions/31.5 Gy/11 fractions/32.5 Gy/11 fractions, 37.5 Gy/15 fractions) was 0.22 (95% CI = 0.10–0.52, *P* < 0.01). The HR for subtype (luminal, HER2, triple-negative) was 2.36 (95% CI = 1.25–4.43, *P* < 0.01). The HR for albumin (<3.9 g or ≥3.9 g/dL) was 0.15 (95% CI = 0.03–0.72, *P* < 0.01).

### Multivariate analysis

Multivariate (stepwise) analysis of the three parameters of LDH, dose, and subtype identified the following significant differences: HR for dose (discontinued, 30 Gy/10 fractions/31.5 Gy/11 fractions/32.5 Gy/11 fractions, 37.5 Gy/15 fractions) was 0.08 (95% CI = 0.02–0.30, *P* < 0.01) and HR for subtype (luminal, HER2, triple-negative) was 2.70 (95% CI = 1.16–6.30, *P* = 0.02).

No significant differences were identified in any other factors. The results are shown in Table 2.

**Table 1.** Patient characteristics (n = 29).

Factor	
Patients (n)	29
Sex	Female
Age (years)	52.2 ± 12.1
*Appearance of brain metastasis*	
At first examination	5 patients
At later stage	24 patients
*Metastases to other organs*	
Yes	27 patients
No	2 patients
*Blood tests*	
WBC (/μL)	8655 ± 5352
RBC (×10^6^/μL)	3.7 ± 0.7
PLT (×10^4^/μL)	26.0 ± 7.7
Total protein (IU/L)	6.5 ± 0.9
Albumin (g/dL)	3.4 ± 0.8
LDH (IU/L)	359.5 ± 245.1
CRP (g/dL)	3.7 ± 5.6
CEA (ng/mL)	14.4 ± 31.9
CA15-3 (U/mL)	101.2 ± 157.9
*Neurological symptoms*	
Yes	11 patients
No	18 patients
*Dose*	
30 Gy/10 fractions, 31.5 Gy/11 fractions, or 32.5 Gy/11 fractions	13 patients
37.5 Gy/15 fractions	14 patients
Discontinued (3 Gy/1 fraction, 15 Gy/5 fractions)	2 patients
*Chemotherapy during whole-brain radiotherapy*	
Yes	5 patients
No	24 patients
*Subtype*	
Luminal	8 patients
HER2	7 patients
Triple-negative	13 patients
Unknown	1 patient
*Additional stereotactic radiotherapy*	
Yes	3 patients
No	26 patients
*Pathology*	
Scirrhous carcinoma	13 patients
Solid tubular carcinoma	7 patients
Papillotubular carcinoma	2 patients
Micropapillary carcinoma	1 patient
Ductal carcinoma in situ	1 patient
Unknown	1 patient

CA15-3, carbohydrate antigen 15-3; CEA, carcinoembryonic antigen; CRP, C-reactive protein; LDH, lactate dehydrogenase; PLT, platelets; RBC, red blood cell count; WBC, white blood cell count.

**Table 2.** Results from Cox’s proportional hazard model.

Factor	Univariate analysis			Multivariate analysis		
	HR	95% CI	*P*	HR	95% CI	*P*
Age	0.99	0.96–1.03	0.70			
Time from onset to brain metastasis	0.29	0.09–1.02	0.05			
Other metastasis when brain metastasis occurred	2.26	0.30–16.9	0.42			
Neurological symptoms	1.10	0.46–2.59	0.82			
WBC (<4000 /µL and ≥4000/µL)	1.23	0.41–3.65	0.71			
RBC (<3.8 × 10^6^/µL and ≥3.8 × 10^6^/µL)	0.60	0.22–1.61	0.31			
PLT (<15.7 × 10^4^/µL and ≥15.7 × 10^4^/µL	8.90	0.93–85.6	0.06			
Stereotactic radiotherapy after whole-brain radiotherapy (performed and not performed)	0.63	0.19–2.17	0.47			
TP (<6.8 mg/dL and ≥6.8 mg/dL)	0.49	0.22–1.01	0.08			
LDH (<230 IU/L and ≥230 IU/L)	2.45	1.03–5.82	0.04	0.27	0.07–1.04	0.06
ALB (<3.9 g/dL and ≥3.9 g/dL)	0.15	0.03–0.72	0.01			
CRP (<0.3 g/dL and ≥0.3 g/dL)	N/A	N/A	N/A			
Dose (discontinued; 30 Gy/10 fractions; 31.5 Gy/11 fractions; 32.5 Gy/11 fractions; 37.5 Gy/15 fractions)	0.22	0.10–0.52	<0.01	0.08	0.02–0.30	<0.01
Subtype (luminal, HER2, TN)	2.36	1.25–4.43	<0.01	2.70	1.16–6.28	<0.01
CEA (<4.3 ng/mL and ≥4.3 ng/mL)	1.16	0.48–2.76	0.74			
CA15-3 (<27 U/mL and ≥27 U/mL)	1.64	0.67–4.06	0.29			
Tumor diameter (<3 cm and ≥3 cm)	0.66	0.08–5.00	0.69			
Number of tumors (5–10 and ≥10)	2.09	0.84–5.21	0.11			
Pathology (scirrhous/others)	1.05	0.42–2.60	0.91			
Chemotherapy (during whole-brain radiotherapy)	1.56	0.57–4.38	0.37			

ALB, albumin; CA15-3, carbohydrate antigen 15-3; CEA, carcinoembryonic antigen; CRP, C-reactive protein; LDH, lactate dehydrogenase; PLT, platelets; RBC, red blood cell count; TP, total protein; WBC, white blood cell count.

## Discussion

The present study investigated prognostic factors for patients who underwent whole-brain radiotherapy as initial treatment for multiple brain metastases (≥5 lesions, no meningeal dissemination) of breast cancer (Table 1). We found that in the case of multiple brain metastases of breast cancer, LDH exhibited a significant value in univariate analysis and a significant boundary in multivariate analysis, suggesting that it may represent a prognostic factor. Studies of small-cell lung cancer patients with metastatic encephalopathy have identified high LDH as a poor prognostic factor (14,15). This has been attributed to high LDH changing the nature of the tumor (14,15) (Table 1).

The present findings also suggested that patients with high LDH experienced increased malignancy. The results of univariate and multivariate analyses also identified subtype as a prognostic factor, showing that patients with the HER2 subtype experience better prognosis. Furthermore, median survival time from the diagnosis of brain metastasis was 7.9 months, and by subtype was 7.1 months for HER2–/HR+, 18.9 months for HER2+/HR+, 13.1 months for HER2+/HR–, and 4.4 months for the triple-negative subtype (2).

Another study reported that patients in good general condition with ≥4 brain metastases who had undergone surgery, stereotactic radiotherapy, or other treatment showed good prognosis for the HER2 subtype (16). However, prognosis was poor in patients who had not undergone systemic chemotherapy (16). On the other hand, the HER2 subtype displays low sensitivity to radiation when it is the primary lesion in breast cancer, and reportedly is responsible for the largest number of recurrences among the three subtypes (luminal, HER2, and triple-negative) (17,18). The reason that HER2 metastatic brain tumors show better prognosis thus cannot be explained by sensitivity to radiation, and it is possible that chemotherapy after whole-brain radiotherapy may have contributed to the response. In fact, HER2 tumors in the present study were given chemotherapy with trastuzumab and pertuzumab after the conclusion of whole-brain radiotherapy and may have responded to this treatment. Univariate and multivariate analyses also identified dose as a prognostic factor. The survival rate was particularly good for patients who received 37.5 Gy/15 fractions. This result indicates the dose-dependency of brain metastases from breast cancer. A dose of 37.5 Gy/15 fractions could thus be recommended.

The reported median survival time for patients with brain metastases of breast cancer who had undergone whole-brain radiotherapy was 14.4 months (19). However, in the present study, median survival time after diagnosis by cranial contrast-enhanced MRI/CT was 135 days (range = 16–2112 days). This was shorter than previously reported, possibly due to the inclusion of different subtypes and treatment methods. Median survival time for the triple-negative subtype was 80 days, compared with 419 days for the luminal subtype. Because of this, patients would likely die before late responses to whole-brain radiotherapy could appear. For the HER2 subtype, on the other hand, median survival time was much longer at 1673 days, and patients would be more likely to survive until the appearance of late response to whole-brain therapy. This result suggested that there is scope to conduct regular whole-brain radiotherapy in patients with triple-negative or luminal-type tumors in cases with multiple brain metastases. For patients with the HER type, careful consideration must be given to the treatment strategy if multiple brain metastases are present. In the JROSG99-1 (Japanese Radiation Oncology Study Group 99-1) study, receiving whole-brain radiotherapy in addition to stereotactic radiotherapy prolonged the OS time of patients in the DS-GPA2.5-4 group with a good prognosis (20). On the basis of that result, whole-brain radiotherapy may be warranted along with stereotactic radiotherapy in HER2-subtype patients. However, because of the risk of cognitive impairment, a desirable treatment policy could be to provide stereotactic radiotherapy after the initial appearance of brain metastases if <10 tumors are present. If whole-brain radiotherapy is also required, using IMRT may be in order to reduce the dose to the hippocampus. The basic dose should ideally be 37.5 Gy/15 fractions. As cognitive impairment after whole-brain radiotherapy is irreversible, the indications must be considered carefully.

The present study also had a number of limitations that need to be considered when interpreting the results. As a retrospective study conducted in a single center, the number of patients was limited. Because Karnofsky performance status was not fully assessed, recursive partition analysis (21), graded prognostic assessment (22), or other evaluations that are helpful for predicting the prognosis of metastatic brain tumors were not able to be performed. In addition, in multivariate analysis we used the stepwise method and the next three parameters—LDH, dose, and subtype—which showed the significant value in single variate analysis as sample size is small. However, in the univariate analysis, platelet value was borderline significant. If the sample size was large and many parameters were used, the result would be changed. Further multicenter prospective studies with larger numbers of patients should be performed in the future.

In conclusion, patients who underwent whole-brain radiotherapy for multiple brain metastases (>5) of breast cancer, HER2-type cancers tended to show better long-term prognosis. A dose of 37.5 Gy/15 fractions also improved long-term prognosis after whole-brain radiotherapy compared with a dose of 30 Gy/10 fractions.

## References

[1] Makita M Iwase T Tada T, et al The site and timing of the first recurrence of breast cancer. Jpn J Breast Cancer 2004; 19:343–351.

[2] Barnholtz-Sloan JS Sloan AE Davis FG, et al Incidence proportions of brain metastases in patients diagnosed (1973 to 2001) in the Metropolitan Detroit Cancer Surveillance System. J Clin Oncol 2004; 22:2865–2872. 15254054 10.1200/JCO.2004.12.149

[3] Andrews DW Scott CB Sperduto PW, et al Whole brain radiation therapy with or without stereotactic radiosurgery boost for patients with one to three brain metastases: phase III results of the RTOG 9508 randomised trial. Lancet 2004; 363:1665–1672. 15158627 10.1016/S0140-6736(04)16250-8

[4] Soffietti R Kocher M Abacioglu UM, et al A European Organisation for Research and Treatment of Cancer phase III trial of adjuvant whole-brain radiotherapy versus observation in patients with one to three brain metastases from solid tumors after surgical resection or radiosurgery: quality-of-life results. J Clin Oncol 2013; 31:65–72. 23213105 10.1200/JCO.2011.41.0639

[5] Kocher M Soffietti R Abacioglu U, et al Adjuvant whole-brain radiotherapy versus observation after radiosurgery or surgical resection of one to three cerebral metastases: results of the EORTC 22952-26001 study. J Clin Oncol 2011; 29:134–141. 21041710 10.1200/JCO.2010.30.1655 PMC3058272

[6] Sahgal A Aoyama H Kocher M, et al Phase 3 trials of stereotactic radiosurgery with or without whole-brain radiation therapy for 1 to 4 brain metastases: individual patient data meta-analysis. Int J Radiat Oncol Biol Phys 2015; 91:710–717. 25752382 10.1016/j.ijrobp.2014.10.024

[7] Yamamoto M Serizawa T Shuto T, et al Stereotactic radiosurgery for patients with multiple brain metastases (JLGK0901): a multi-institutional prospective observational study. Lancet Oncol 2014; 15:387–395. 24621620 10.1016/S1470-2045(14)70061-0

[8] Aoyama H Tago M Kato N, et al Neurocognitive function of patients with brain metastasis who received either whole brain radiotherapy plus stereotactic radiosurgery or radiosurgery alone. Int J Radiat Oncol Biol Phys 2007; 68:1388–1395. 17674975 10.1016/j.ijrobp.2007.03.048

[9] Brown PD Pugh S Laack NN, et al Memantine for the prevention of cognitive dysfunction in patients receiving whole-brain radiotherapy: a randomized, double-blind, placebo-controlled trial. Neuro Oncol 2013; 15:1429–1437. 23956241 10.1093/neuonc/not114 PMC3779047

[10] Chang EL Wefel JS Hess KR, et al Neurocognition in patients with brain metastases treated with radiosurgery or radiosurgery plus whole-brain irradiation: a randomised controlled trial. Lancet Oncol 2009; 10:1037–1044. 19801201 10.1016/S1470-2045(09)70263-3

[11] Brown PD Jaeckle K Ballman KV, et al Effect of radiosurgery alone vs radiosurgery with whole brain radiation therapy on cognitive function in patients with 1 to 3 brain metastases: a randomized clinical trial. JAMA 2016; 316:401–409. 27458945 10.1001/jama.2016.9839 PMC5313044

[12] Harth S Abo-Madyan Y Zheng L, et al Estimation of intracranial failure risk following hippocampal-sparing whole brain radiotherapy. Radiother Oncol 2013; 109:152–158. 24100152 10.1016/j.radonc.2013.09.009

[13] Gondi V Pugh SL Tome WA, et al Preservation of memory with conformal avoidance of the hippocampal neural stem-cell compartment during whole-brain radiotherapy for brain metastases (RTOG 0933): a phase II multi-institutional trial. J Clin Oncol 2014; 32:3810–3816. 25349290 10.1200/JCO.2014.57.2909 PMC4239303

[14] Anami S Doi H Nakamatsu K, et al Serum lactate dehydrogenase predicts survival in small-cell lung cancer patients with brain metastases that were treated with whole-brain radiotherapy. J Radiat Res 2019; 60:257–263. 30576550 10.1093/jrr/rry107 PMC6430245

[15] Wen Q Meng X Xie P, et al Evaluation of factors associated with platinum-sensitivity status and survival in limited-stage small cell lung cancer patients treated with chemoradiotherapy. Oncotarget 2017; 8:81405–81418. 29113400 10.18632/oncotarget.19073 PMC5655295

[16] Kuba S Ishida M Nakamura Y, et al Treatment and prognosis of breast cancer patients with brain metastases according to intrinsic subtype. Jpn J Clin Oncol 2014; 44:1025–1031. 25156682 10.1093/jjco/hyu126

[17] Demirci S Broadwater G Marks LB, et al Breast conservation therapy: the influence of molecular subtype and margins. Int J Radiat Oncol Biol Phys 2012; 83:814–820. 22208971 10.1016/j.ijrobp.2011.09.001

[18] Albert JM Gonzalez-Angulo AM Guray M, et al Estrogen/progesterone receptor negativity and HER2 positivity predict locoregional recurrence in patients with T1a,bN0 breast cancer. Int J Radiat Oncol Biol Phys 2010; 77:1296–1302. 20472353 10.1016/j.ijrobp.2009.12.011

[19] Ramakrishna N Temin S Chandarlapaty S, et al Recommendations on disease management for patients with advanced human epidermal growth factor receptor 2-positive breast cancer and brain metastases: American Society of Clinical Oncology clinical practice guideline. J Clin Oncol 2014; 32:2100–2108. 24799487 10.1200/JCO.2013.54.0955 PMC6366342

[20] Aoyama H Tago M Shirato H. Stereotactic radiosurgery with or without whole-brain radiotherapy for brain metastases: secondary analysis of the JROSG 99-1 randomized clinical trial. JAMA Oncol 2015; 1:457–464. 26181254 10.1001/jamaoncol.2015.1145

[21] Gaspar L Scott C Rotman M, et al Recursive partitioning analysis (RPA) of prognostic factors in three Radiation Therapy Oncology Group (RTOG) brain metastases trials. Int J Radiat Oncol Biol Phys 1997; 37:745–751. 9128946 10.1016/s0360-3016(96)00619-0

[22] Sperduto PW Kased N Roberge D, et al Summary report on the graded prognostic assessment: an accurate and facile diagnosis-specific tool to estimate survival for patients with brain metastases. J Clin Oncol 2012; 30:419–425. 22203767 10.1200/JCO.2011.38.0527 PMC3269967

